# Neuraminidase and Hemagglutinin Matching Patterns of a Highly Pathogenic Avian and Two Pandemic H1N1 Influenza A Viruses

**DOI:** 10.1371/journal.pone.0009167

**Published:** 2010-02-11

**Authors:** Yonghui Zhang, Xiaojing Lin, Guoqin Wang, Jianfang Zhou, Jian Lu, Honglan Zhao, Fengwei Zhang, Jia Wu, Chunqiong Xu, Ning Du, Zi Li, Ye Zhang, Xiaoyi Wang, Shengli Bi, Yuelong Shu, Hongning Zhou, Wenjie Tan, Xiaobing Wu, Zhihui Chen, Yue Wang

**Affiliations:** 1 Institute of Pathogens and Vectors, Dali University, Dali, China; 2 Department of Infectious Disease, Changhai Hospital, Second Military Medical University, Shanghai, China; 3 State Key Laboratory for Molecular Virology and Genetic Engineering, National Institute for Viral Disease Control and Prevention, Chinese Center for Disease Control and Prevention, Beijing, China; 4 Kitasato Clinical Research Center, School of Medicine, Kitasato University, Sagamihara, Japan; Tsinghua University, China

## Abstract

**Background:**

Influenza A virus displays strong reassortment characteristics, which enable it to achieve adaptation in human infection. Surveying the reassortment and virulence of novel viruses is important in the prevention and control of an influenza pandemic. Meanwhile, studying the mechanism of reassortment may accelerate the development of anti-influenza strategies.

**Methodology/Principal Findings:**

The hemagglutinin (HA) and neuraminidase (NA) matching patterns of two pandemic H1N1 viruses (the 1918 and current 2009 strains) and a highly pathogenic avian influenza A virus (H5N1) were studied using a pseudotyped particle (pp) system. Our data showed that four of the six chimeric HA/NA combinations could produce infectious pps, and that some of the chimeric pps had greater infectivity than did their ancestors, raising the possibility of reassortment among these viruses. The NA of H5N1 (A/Anhui/1/2005) could hardly reassort with the HAs of the two H1N1 viruses. Many biological characteristics of HA and NA, including infectivity, hemagglutinating ability, and NA activity, are dependent on their matching pattern.

**Conclusions/Significance:**

Our data suggest the existence of an interaction between HA and NA, and the HA NA matching pattern is critical for valid viral reassortment.

## Introduction

Influenza A virus has caused several epidemics, including the catastrophic H1N1 Spanish flu of 1918 (more than 50 million deaths globally), the H2N2 Asian flu of 1957 (more than 1 million deaths globally), and the H3N2 Hong Kong flu of 1968 (∼0.5 million deaths globally) [1]–[3]. In comparison, the current outbreak of a novel H1N1 viral strain, which has become a major global issue since April of 2009, has affected 177 countries, including more than 182,166 people, and caused 1,799 deaths to date [4]. Simultaneously, a highly pathogenic avian influenza virus, H5N1, has been circulating in Eurasia for more than a decade and has spread to more than 60 countries; thus far, it has infected 438 humans and killed 262 [5]. Although there are only rare reports of human- to-human H5N1 transmission to date, its high lethality has raised considerable concern worldwide.

Influenza virus A is a genus in the *Orthomyxoviridae* family; its genome contains eight negative single-stranded RNA segments that encode eleven proteins (HA, NA, NP, M1, M2, NS1, NEP, PA, PB1, PB1-F2, and PB2) and is subtyped based on 16 hemagglutinin (HA) and nine neuraminidase (NA) envelope proteins [1]. Reassortment of the eight RNA segments may produce additional novel viruses, at least theoretically [1]–[3]. Influenza A viruses are believed to enter host cells via HA binding to sialic acid receptors on the cell surface; the binding affinity of HA to sialic acid is believed to account in part for the host specificity of several influenza A viral subtypes [1], [6], [7]. Human viruses known to be capable of tropism preferentially bind to sialic acid linked to galactose by α-2,6 linkages, while avian viruses of this type tend to bind to α-2,3 linkages [7]. Many animals, including swine, chickens, and humans, have both α-2,3 and α-2,6 linkages on their epithelial cells, which may serve as a “mixing vessel” for the genesis of new viral types through co-infection [2], [3], [8]. In fact, genetic studies of the current H1N1 strain have suggested that the virus contains a unique combination of gene segments from both North American and Eurasian swine lineages, of which PB2, PA, PB1, HA, NP, and NS are derived from a North American swine lineage (triple reassortment), while NA and M are derived from a Eurasian swine lineage [9], [10]. In other words, the current pandemic H1N1 virus carries a complex genetic reassortment of previously prevalent viral strains [9], [10].

Influenza A viral subtypes, including Spanish influenza H1N1, the current pandemic H1N1 viruses, and influenza A viral subtype HPAI H5N1, are catastrophic pathogens for humans [4], [11]–[14]. Although the Spanish influenza H1N1 virus ultimately disappeared from surveillance, it's possible that the strain remains on Earth, and the reassortment of two or more catastrophic viruses may present a challenge to human survival. Although we understand the general mechanisms by which new influenza viruses emerge, our basic knowledge of how these viruses reassort and acquire human pandemic potential is limited, and our molecular understanding of the virus and host factors involved in successful transmission and spread is rudimentary.

Increasing industrialization and travel, and the huge populations of many influenza viral reservoir animals will likely accelerate the emergence of novel viruses, especially those viruses that readily reassort. Surveying new viral strains is one aspect of influenza virus control and prevention. Thus, research on possible reassortment among the HPAI H5N1, H1N1 2009, and H1N1 1918 viruses is needed with regard to therapeutic target and preventive strategy development. In this study, the HA and NA matching patterns and biological properties of two pandemic H1N1 strains (Spanish H1N1 1918 and the current strain) and a HPAI H5N1 strain were studied using a newly established relative influenza virus pseudotyped particle (pp) system. Our study provides important biological information about these viruses.

## Materials and Methods

### Cell Culture

A549 cells and human embryonic kidney (HEK) 293T cells (American Type Culture Collection, Manassas, VA) were grown in Dulbecco's modified essential medium (DMEM; Invitrogen, Carlsbad, CA) supplemented with 10% fetal bovine serum.

### Plasmid Construction

cDNA fragments encoding the full-length HA of A/South Carolina/1/1918 (GenBank Acc. No. AF117241) and A/California/05/2009 (GenBank Acc. No. FJ966952) and the full-length NA of A/Brevig_Mission/1/1918 (GenBank Acc. No. AF250356) and A/Ohio/07/2009 (GenBank Acc. No. FJ969534) were synthesized and inserted into the vector pVRC (Liuhetong Inc., Beijing, China) to produce the constructs 1918H1, 09H1, 1918N1, and 09N1, respectively [15], [16].

Similarly, cDNA fragments encoding the full-length HA and NA from H5N1 (A/Anhui/1/2005, GenBank Acc. No. DQ371928) were amplified and cloned into the vector pVRC; they are referred to as AH H5 and AH N1, respectively.

### Pp Production and Quantification

Pps were produced by transfecting 293T cells with four plasmids: an HA expression plasmid, an NA expression plasmid, a Gag-pol-encoding plasmid, and a reporter plasmid. At 72 h post-transfection, pps were harvested from the supernatants of the transfected cells by filtration through a 0.45-µm Durapore PVDF membrane filter (Millipore Ireland, Cork, Ireland). For quantification of pps, purified pps were treated with DNase/RNase to eliminate the possible contamination of DNA/RNA, and CMV-GFP RNA in pps was extracted by QIAamp UltraSens Virus Kit (QIAGEN, CA). The amount of CMV-GFP RNA in each pps was quantified by qRT-PCR using forward primer: CCCGTGAGTCAAACCGCTAT, reverse primer: GTGATGCGGTTTTGGCAGTA, and probe: FAM-CCACGCCCATTGATG-NFQ in an ABI 7500 Fast (Applied Biosystem, Foster City, CA) with One Step PrimeScript RT-PCR Kit (Takara, Japan), by which all pps were normalized to the same cycle threshold (Ct) value to 12∼13 prior to the following experiments including infection assay, hemagglutination assay and NA activity detection and western blot analysis of the HA and NA combinations.

### Infection Assay

The pps infection assay was performed as reported previously [15], [16]. The naïve H1N1 virus HA0 must be matured by TPCK-trypsin cleavage to form its functional subunits HA1 and HA2; using optimal conditions for pp infection, normalized pps were pre-treated with TPCK-trypsin at a final concentration of 40 µg/mL at 37°C for 1 h before infection. A549 cells (5−10×10^3^) were seeded in a 96-well plate one day prior to infection. The normalized pps were diluted 1∶1 in 100 µL of DMEM. For the infection process, the medium in the plate was replaced with the diluted pps, allowed to stand for 4 h, and then replaced with DMEM supplemented with 3% FBS. At 72 h post-infection, the infected cells were rinsed twice with phosphate-buffered saline (PBS). The number of GFP reporter-positive cells was determined by FACS (BD FACSAria, BD Biosciences, Franklin Lakes, NJ).

### Hemagglutination Assay

To determine the hemagglutinating activity of the pps, a hemagglutination assay was performed. The normalized pp samples were two-fold diluted serially in PBS at 50 µL per well. Next, 50-µL aliquots of 1% turkey red blood cells were added to each well and left to stand for 30 min; hemagglutination was then scored.

### NA Activity Detection

To detect the level of NA activity in each HA and NA combination, an NA activity assay was performed using the NA-Star Influenza Neuraminidase Inhibitor Resistance Detection Kit (Applied Biosystems, Foster City, CA). All reagents were prepared according to the manufacturer's instructions. For each sample, normalized pps were diluted 1∶1 in NA-Star Assay buffer (the same concentration as in the infection assay) then incubated with 10 µL of NA-Star substrate for 30 min at room temperature. The samples were then analyzed using a luminometer (2103 Envision Multilabel Reader, PerkinElmer, Waltham, MA).

### Western Blot and Immunocellular Staining Analysis of the HA and NA Combinations

To assess the existence of HA and NA in the pps, 2 mL normalized pp combination were purified and concentrated to 20 µL using PEG-it™ Virus Precipitation Solution (System Bioscience, Mountain View, CA). To assess the HA and NA expression in 293T producer cells, after pp harvest, the 293T cells in 6-well plate were washed twice by PBS to remove residual surpernatant and lysed in 500 µL of reporter lysis buffer (Promega, WI) per well and centrifugated to remove cell debris. 20 µL of each sample (prepared pps and cell lysates) were loaded with 4× LDS sample buffer (Invitrogen, Carlsbad, CA) and heated to 100°C for 5 min and applied to electrophoresis using a 12% NuPage gel (Invitrogen, Carlsbad, CA). After electrophoresis, the gel was semi-dry transferred to a PVDF membrane (Pall Corp., Port Washington, NY). For HA and NA identification, the membrane was blocked overnight in skim milk at 4°C. Following incubation in primary antibody to HAs (antibodies to 09H1 and 1918H1 were provided by Sino Biological Inc, Beijing, China; antibody to AH H5 was provided by Dr. Mifang Liang [17]) and primary antibody to NAs (antibodies to 09N1, 1918N1 and AH N1 were raised by our laboratory and purchased from Abcam, Cambridge, MA) for 1.5 h at room temperature, the membrane was washed three times in PBS. Next, the membrane was incubated in corresponding biotinylated secondary antibody (Vector Laboratories, CA) for 20 min at room temperature, washed three times in PBS, and visualized by DAB SUBSTRATE KIT (Vector Laboratories, CA).

To examine the HAs and NAs expression of all 9 combinations, 293T producer cells in 6-well plate were washed twice with PBS and subsequently fixed them with 3.7% formaldehyde in PBS for 1 hour at room temperature. After washing the cells again with PBS, we permeabilized the cells with 0.1% Tween 20 in PBS for 1 hour, blocked the cells with PBS containing 2% normal serum for 1 hour, and incubated the cells with primary antibodies for 2 hours at room temperature. We washed the cells 3 times with PBS and then incubated the cells with FITC-conjugated secondary antibodies (Dako, Carpinteria, CA) for1 hour at room temperature. After washing the cells with ice-cold PBS, we coated the cells with fluorescent mounting medium (Dako), covered them with glass coverslips, and observed the stained cells with a Nikon Fluorescence Microscopy (Nikon, Japan).

### Data Analysis

Significant differences were evaluated using a two-tailed Fisher's exact test (SPSS, release 12.1; SPSS Inc., Chicago, IL). Differences were deemed to be statistically significant at p<0.05.

## Results

### Four of the Six Chimeric HA/NA Combinations Produced Infectious Pps

**Figure 1:**
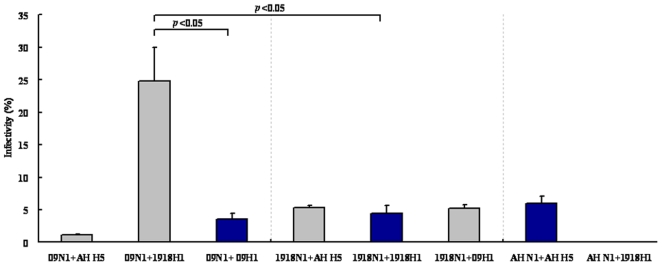
Infectivity assay of all combinations. Infectivity of normalized pps with various HA/NA combinations; infectivity is presented as the Mean± SD percentage of infected cells from 3 repeats. All pps were grouped by NA; 2009 H1N1, 1918 H1N1, and H5N1 (A/Anhui/1/2005) are abbreviated as 09, 1918, and AH, respectively.

HA and NA are envelope proteins located on the viral surface that co-mediate the first stages of viral entry; thus, to achieve successful infection for a given viral strain, the naïve HA and NA should match each other well. Unlike the hepatitis C virus, where the envelope proteins E1 and E2 form a heterodimer for viral entry [18], HA and NA seem to be discrete on the viral surface, at a ratio of about 4∶1 [1], so it appears reasonable that the HA should match the NAs in addition to its own spousal NA to reassort [19], [20]. We demonstrated previously that the chimeric combination of 09 H1+1918 N1 and 1918 H1+09 N1 could form infectious pps [15]. To further investigate whether the HA matches the NA, rather than that belonging to the same subtype, and to evaluate whether the above mentioned three catastrophic influenza viruses can undergo reassortment, we generated pps using the following combinations: 09N1+09H1, 1918N1+1918H1, and AH N1+AH H5, as well as 09N1+AH H5, 09N1+1918H1, 1918N1+AH H5, 1918N1+09H1, AN N1+1918H1, and AH N1+09H1. The combination of 09N1+09H1, 1918N1+1918H1, and AH N1+AH H5 resulted in the formation of infectious pps, with infectivities of 3.60±0.85, 4.45±1.20, and 6.05±1.06%, respectively (Fig. 1; dark blue bars). As expected, the combination of 09N1+AH H5, 09N1+1918H1, 1918N1+AH H5, and 1918N1+09H1 also resulted in highly infectious pps, with infectivities of 1.15±0.07, 24.85±5.16, 5.35±0.35, and 5.15±0.64%, respectively (Fig. 1). Of the pps containing 09N1, the combination of 09N1+1918H1 had greater infectivity than the ancestor 09N1+09H1 (p<0.05). The pps containing 1918N1, 1918N1+AH H5, and 1918N1+09H1 also showed greater infectivity than their ancestor, 1918N1+1918H1. 09N1+1918H1 also showed greater infectivity than another ancestor, 1918N1+1918H1 (p<0.05). Take together, these data suggest that valid reassortment may occur not only between viruses belonging to the same subtype (H1N1), but also between viruses belonging to different subtypes (H1N1 and H5N1); furthermore, some viruses, the products of reassortment, may have greater infectivity than their ancestors.

Unexpectedly, of the combinations involving AH N1, only the naïve combination AH N1+AH H5 produced infectious pps, while AH N1+09H1 and AH N1+1918H1 displayed very low infectivity (similar to that of HA alone), suggesting that the NA of H5N1 did not match the HA belonging to H1N1 well (Fig. 1). In conclusion, our data indicate that four of the six chimeric HA/NA combinations could produce infectious pps, and that some of the chimeric pps had greater infectivity than their ancestors, raising concern about the possible reassortment of these viruses. Meanwhile, the NA of H5N1 (A/Anhui/1/2005) was apparently hard to reassort with the HAs of two other H1N1 viruses.

### HA/NA Expression Analysis

**Figure 2:**
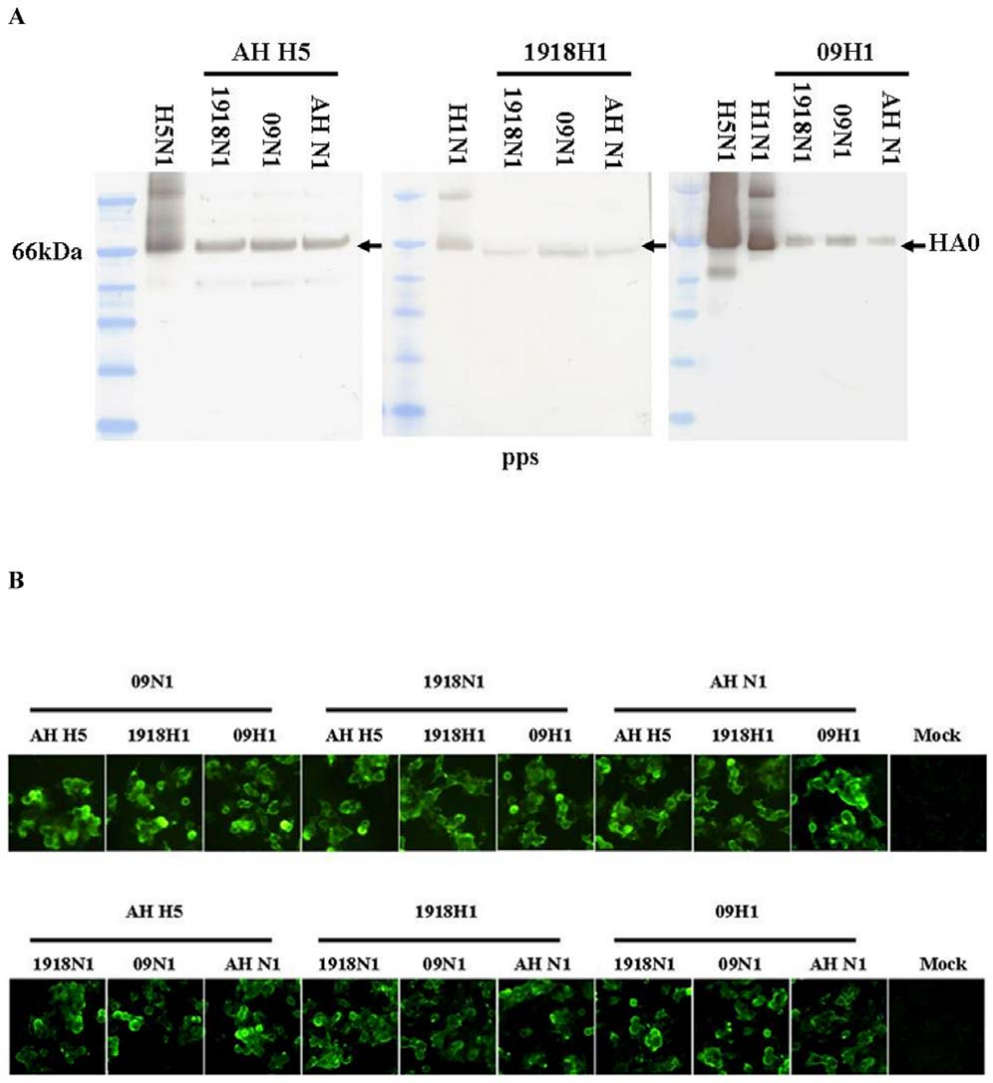
HA/NA expression analysis. A. Western blot of HA. HA of each pp combination was blotted with purified 300ng wild viruses as positive control (H5N1: A/Vietnam/1194/2005; H1N1: A/Carifornia/7/2009). B. Immunocellular staining of HA and NA on pp producer 293T cells. HA (bottom) and NA (upper). Mock, normal 293T cell lysate.

To eliminate the possibility of differences in HA and NA expression efficiency among all of the combinations and to further assess the biological features of the HAs and NAs on all virions, both pps and pp producer cells were subjected to Western blot analysis. All primary antibodies for HAs could recognize the linearizes HA0s well. Of the pps derived from AH H5, 1918H1 and 09H1 combined with 1918N1, 09N1, and AH N1, HAs showed similar amount within each subgroup, all HAs displayed similar molecular weight to purified wild influ viruses H5N1 and H1N1. In the subgroup of the 1918H1 combined with 1918N1, 09N1, and AH N1, 1918H1 was relatively weak than other two HAs, considering that 09N1+1918H1 hit the highest infectivity, the difference in the “amount” of 1918H1 should be due to the affinity of the primary antibody (Fig. 2A).

Of the NAs western blot analysis, we tired as many as ten strain specific and non-specific primary antibodies, only in the cellular lysate transfected by AH N1 could be recognized and visualized by a Rabbit polyclonal to Avian Influenza A Neuraminidase (Abcam, ab70759), this antibody could not make the AH N1 of the purified wild H5N1 virus visible even the loading amount was five times higher than that in HA detection (data not showed). This may be explained by that many antibodies might only bind to conformational epitope in NA and the NA amount in the virons or combinations was low.

To confirm the HAs and NAs expression in pp producer 293T cells, after the pp harvested, 293T cells in 6-well plate were subjected to immunocellular staining. For HAs expression, all antibodies we used in western blot analysis could recognize the HAs well, HAs in all combinations showed similar character in subcellular location and expression efficiency (Fig. 2B bottom). For NAs expression, many antibodies including Rabbit polyclonal to Avian Influenza A Neuraminidase (Abcam, ab70759) could recognize all NAs well, they displayed similar character in subcellular location and expression efficiency (Fig. 2B upper).

In conclusion, although we failed to detect the NAs in pps, our immunocellular staining showed that all HAs and NAs were expressed equally in pp producer cells and our normalized pps showed similar amount of HAs in all combinations, the difference of infectivity among all combinations should be due to the HA NA matching patterns.

### Hemagglutination Among the Combinations

**Figure 3:**
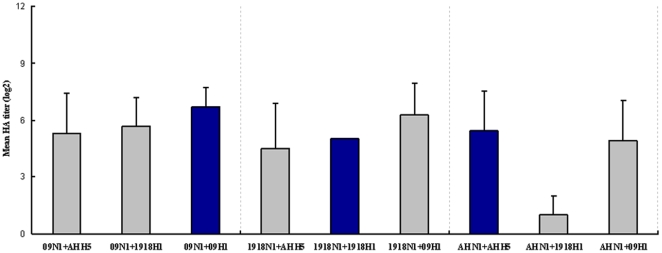
Hemagglination assay of various pps. All pps were two-fold diluted serially in 96-well plate. The hemagglutinating ability was expression as the mean HA titer (log_2_ HA units/50 µl) of each pp, n = 3.

To further characterize the pps and to confirm the existence of HA activity in the combinations, the normalized pp samples generated from the nine combinations were subjected to a hemagglutination assay. All pps were diluted two-fold serially in this assay. The hemagglutinating abilities (log_2_) of 09N1+AH H5, 09N1+1918H1, and 09N1+09H1 (all containing NA from the 2009 H1N1 strain) were 5.3±2.1, 5.7±1.5, and 6.7±0.9, respectively (Fig. 3); the naïve combination 09N1+09H1 displayed the highest level of hemagglutination. Interestingly, the 09N1+1918H1 pps, which showed the greatest infectivity, did not display the highest level of hemagglutination (Fig. 3 and Fig. 1). The hemagglutination abilities (log_2_) of 1918N1+AH H5, 1918N1+1918H1, and 1918N1+09H1 (all containing NA from the 1918 H1N1 strain) were 4.5±2.4, 5±0, and 6.3±1.7, respectively (Fig. 3). The hemagglutinating abilities (log_2_) of AH N1+AH H5, AH N1+1918H1, and AH N1+09H1 (all containing NA from the H5N1 strain) were 5.4±2.1, 1±1, and 4.9±2.1, respectively (Fig. 3); For AH N1+1918H1 and AH N1+09H1 combinations which displayed very low infectivity, although AH N1+1918H1 showed the lowest hemagglutinating ability, AH N1+09H1 showed fair high hemagglutinating ability (Fig. 3). Taken together, our data suggested that hemagglutinating ability seemed to have no relationship with infectivity.

### NA Activity in the Pps

**Figure 4:**
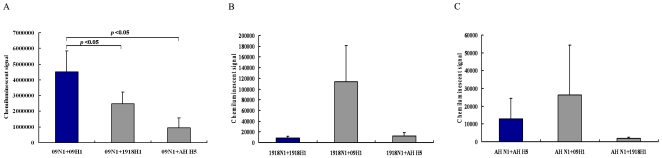
NA activity in the various pps, NA activity is presented as the Mean± SD from 3 repeats. A. NA activity of 09N1 combined with 09H1, 1918H1, and AH H1. B. NA activity of 1918N1 combined with 1918H1, 09H1, and AH H1. C. NA activity of AH N1 combined with AH H1, 09H1, and 1918H1.

To determine the level of NA activity in the pps, normalized pp samples generated from the nine combinations were subjected to NA activity assays. NA activity in the pps containing the NA from the 2009 H1N1, 1918 H1N1, and H5N1 strains was 2645133±1754985, 45027±61835, and 13684±18463 chemiluminescent units, respectively, indicating a tendency from high to low. NA activity in the pps containing 2009NA was significantly higher than in those containing 1918NA and H5N1 NA (p = 0.0022 and 0.0019, respectively; Fig. 4). This tendency was consistent with the degree of infectivity (Fig. 1). Focusing on the groups categorized by the three NAs, in the pp group containing 2009NA, the NA activities of 09N1+09H1, 09N1+1918H1, and 09N1+AH H5 were 4511453±1334912, 2477080±743487, and 946867±625996 chemiluminescent units, respectively. Thus, the level of NA activity in 09N1+09H1 was significantly higher than that in 09N1+1918H1 or 09N1+AH H5 (p = 0.049 and 0.013, respectively; Fig. 4A). In the pp group containing 1918NA, the level of NA activity in 1918N1+1918H1, 1918N1+09H1, and 1918N1+AH H5 was 8400±2892, 113920±67540, and 12760±5557 chemiluminescent units, respectively. Thus, the NA activity of 1918N1+09H1 was higher than that of its ancestor 1918N1+1918H1 (Fig. 4B). In the pp group containing AH NA, the NA activities of AH N1+AH H5, AH N1+09H1, and AH N1+1918 H1 were 12827±11499, 26333±27953, and 1893±583 chemiluminescent units, respectively. Thus, the NA activity of AH N1+09H1 was higher than that of its ancestor AH N1+AH H5 (Fig. 4C). Taken together, NA activity varied immensely between the strains. Furthermore, the level of NA activity showed a spouse-dependent tendency; HA appeared to be able to raise the activity of all of the NAs, especially the HA from the 2009 H1N1 strain.

### Primary Sequence Analysis of Three HA/NA Combinations

**Figure 5:**
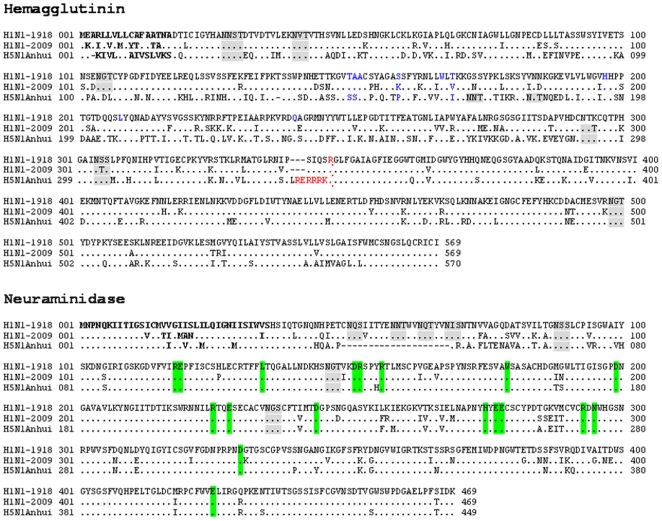
Primary sequence alignment of the HAs and NAs. The H1N1 1918 strain was used as a standard. Those residues that are identical in the 1918 strain are shown by “.” The signal peptide is indicated in bold. The aas marked in red represent the cleavage site of the HA precursor, linking the functional HA1 and HA2 domains, while those marked in blue indicate the binding sites for the sialic acid receptor. The gray boxes indicate potential glycosylation sites, as predicted from the sequence, while the green box indicates conserved active site residues from the influenza virus NA.

To standardize the research, amino acid sequences, we aligned the primary sequences of the HAs from A/South Carolina/1/1918 (GenBank Acc. No. AF117241), A/California/05/2009 (GenBank Acc. No. FJ966952), and A/Anhui/1/2005 (GenBank Acc. No. DQ371928) and of the NAs from A/Brevig_Mission/1/1918 (GenBank Acc. No. AF250356), A/Anhui/1/2005 (GenBank Acc. No. DQ371928), and A/Ohio/07/2009 (GenBank Acc. No. FJ969534) using the HA and NA from the 1918 strain as standards. Residues identical to those in the 1918 strain were replaced by “.”; the aas in red represent the cleavage site of the HA precursor, linking the functional HA1 and HA2 domains; bold indicates the signal peptide; blue indicates the sialic acid receptor binding sites; gray boxes indicate potential glycosylation sites as predicted from the HA1 sequence; and the green box indicates the residues corresponding to the active site of NA (Fig. 5).

Of the 569 aas in HA, 79 (13.88%) and 200 (35.15%) aa differences were detected in the HAs of the 2009 and H5N1 strains compared to those of the 1918 strain, respectively (Fig. 5). Although the aas showed considerable variation in the H1N1 strains, the HA of H5N1 displayed similar glycosylation and receptor binding sites to the H1N1 strains. Together with the fact that the HA from H5N1 could match not only its naïve NA but also the NAs from the 2009 and 1918 strains, the HA of H5N1 would be expected to share biological features, including an affinity for NA, with that of the two H1N1 strains.

Of the 469 aas in NA, 59 (12.85%) of those in 09NA and 52 (11.09%) of those in the NA from H5N1 were different from those in the 1918 strain (Fig. 5). Although the NA of H5N1 showed less variation than the H1N1 strains, as many as 20 aas, at positions 49–68, were absent (Fig. 5).

## Discussion

The high mutation rate in minus-strand RNA viruses, the ability of gene segments to reassort, the transmission route, and the huge pool of influenza viruses in birds and mammals ensure their continuously changing behavior [21]. Thus, the survey of reassortment and virulence is a key aspect in the prevention and control of influenza pandemics. The surface proteins of influenza viruses, HA and NA, play important roles in virulence, host specificity, and the human immune response [1]. In this study, we evaluated the HA and NA matching patterns of two pandemic H1N1 viruses (1918 and the current 2009 strain) and a highly pathogenic avian influenza A virus (H5N1) using a retroviral pp system. Our data show that four of the six chimeric HA/NA combinations produced infectious pps and that some of the chimeric pps had greater infectivity than did their ancestors, raising the possibility of reassortment in these viruses.

Influenza A viruses belong to the family *Orthomyxoviridae*. On the basis of the antigenicity of their HA and NA molecules, they are classified into 16 HA subtypes (H1-H16) and nine NA subtypes (N1-N9) [1]. Thus, theoretically there should be 144 subtypes. However, historically, only a few subtypes of influenza virus have ever produced epidemics or pandemics in humans, including H1N1, H2N2, and H3N2 [2], [3]. These pandemic viruses showed clear features of genetic reassorment; eight gene fragments were originally derived from avian viruses [22], while HA, PB1, and/or NA alone were derived from avian viruses [23], [24]. Thus, HA and NA play important roles in the emergence and global transmission of novel viruses in humans. Recently, HPAI H5N1 [25], [26] was directly transmitted to humans, like the avian flu viruses H7N7 [27], H9N2 [28], and H5N2 [29]; however, the human-to-human transmission of these viruses is limited [2], [3]. The above evidence suggests certain limiting factors among the subtypes of viruses regarding valid reassorments and pandemics. Many factors can influence influenza virus transmission and spread [30]. Our study demonstrates that N1 from H5N1 did not match well with the two HAs from H1N1, indicating that the HA/NA matching pattern is an important factor in determining the strain's virological properties. The continuing outbreaks in poultry and birds caused by H5N1 with the 20-aa deletion in the NA stalk region, which emerged in 1999 [31], suggest that this NA prefers avian hosts. Our data show that the naïve combination of AH N1+AH H5 displayed strong infectivity in A549 cells, seemingly removing the possibility that the weak infectivity of the AH N1+1918H1 and AH N1+09H1 combinations was not caused by species restrictions. The deletion of aas 49–68 in AH N1 may be important in HAs NAs interaction, but a study showed that deletions of up to 28 amino acids and insertions of up to 41 amino acids in the stalk region did not abolish formation of infectious progeny virus [32]. So AH N1 might has strong species restriction and this restriction may limit the H5N1 transmission among human.

For the NAs detection in pps, although we failed to demonstrate the incorporation of HAs into nine pps, our NA activity assay showed that all NAs did display enzyme activity, the infectivity assay showed that NAs did participate in the infection, and the immunocellular staining showed that all NAs expressed at the same level. In fact, study showed that the NA amount is not more than 1/40 of the HA amount in wild influ virus [33], not like the statement NA/HA is 1/4 [1], so the failure of NAs detection may also be partially due to the low amount of NAs in pps and we believe that the variation in infectivity should be not due to the difference of NAs incorporation. There are many possible hypotheses to explain in the increase and decrease NA activity including the incorporated amount of NA in each kind of pps and the interaction between a given HA and a certain NA, we will try to explain this issue in our following experiments.
